# Sunflower Oil Fortified with Vitamins D and A and Sunflower Lecithin Ameliorated Scopolamine-Induced Cognitive Dysfunction in Mice and Exploration of the Underlying Protective Pathways

**DOI:** 10.3390/nu17030553

**Published:** 2025-01-31

**Authors:** Xue Tang, Chengkai Zhu, Tristan C. Liu, Rongxiang Zhu, Guoliang Deng, Peng Zhou, Dasong Liu

**Affiliations:** 1 School of Food Science and Technology, Jiangnan University, Wuxi 214122, China; tangxue@jiangnan.edu.cn (X.T.); 6220111270@stu.jiangnan.edu.cn (C.Z.); 6210113263@stu.jiangnan.edu.cn (R.Z.); 6210112142@stu.jiangnan.edu.cn (G.D.); 2 National Engineering Research Center for Functional Food, Jiangnan University, Wuxi 214122, China; zhoupeng@jiangnan.edu.cn (P.Z.); liudasong@jiangnan.edu.cn (D.L.)

**Keywords:** sunflower lecithin, vitamin D, vitamin A, cognitive dysfunction, PI3K-AKT signaling pathway, cholinergic pathway, folate biosynthesis pathway

## Abstract

The incidence of cognitive disorders is increasing globally, with a reported prevalence of over 50 million individuals affected, and current interventions offer limited efficacy. This study investigates the effects of sunflower oil fortified with sunflower lecithin, vitamin D, and vitamin A on scopolamine-induced cognitive dysfunction in mice and explores the underlying mechanisms. The incidence of cognitive disorders, such as Alzheimer’s disease, is increasing yearly, and current interventions offer limited efficacy. Therefore, this research aims to evaluate the cognitive improvement effects of the three added functional factors on mice with learning and memory impairments, along with the associated molecular mechanisms. Behavioral tests, biochemical assays, and real-time quantitative polymerase chain reaction (RT-qPCR) were utilized to examine the intervention effects of these functional factors on scopolamine-induced cognitive impairment in mice. The results revealed that the groups treated with sunflower lecithin and vitamin D significantly enhanced the mice’s exploratory behavior, working memory, and spatial memory, with increases of 1.6 times and 4.5 times, respectively, in the open field and novel object recognition tests (VD group). Additionally, these treatments reduced levels of inflammatory markers and IL-6, increased antioxidant GSH levels, and decreased oxidative stress marker MDA levels, with all effects showing significant differences (*p* < 0.01). The effects were further enhanced when vitamin A was combined with these treatments. Transcriptomic analysis demonstrated that the intervention groups had markedly improved learning and memory abilities through upregulation of key gene expression levels in the PI3K-AKT signaling pathway, cholinergic pathway, and folate biosynthesis pathway. These findings provide a theoretical basis for the development of nutritionally fortified edible oils with added sunflower lecithin, vitamin D, and vitamin A, which may help prevent and ameliorate cognitive disorders.

## 1. Introduction

Cognitive disorders encompass a range of conditions that impair memory, language, thinking, and judgment, including Alzheimer’s disease and other forms of dementia. With the global aging population, the prevalence of cognitive disorders is rising annually, imposing significant economic and psychological burdens on society and families [1]. Research indicates that unhealthy lifestyles, such as chronic stress, poor diet, lack of exercise, and insufficient sleep, are major contributing factors to cognitive disorders [2]. Despite extensive research into the prevention and treatment of cognitive disorders, effective interventions remain limited, highlighting the need for novel strategies. As research has shown, the density of oils and the composition of fatty acids play a significant role in influencing brain health [3]. Inappropriate oil intake, particularly excessive consumption of saturated fatty acids, may lead to brain inflammation and exacerbate cognitive impairment [4]. Therefore, choosing oils with a balanced fatty acid composition is emerging as a potential strategy for preventing and alleviating cognitive disorders. For example, oils rich in unsaturated fatty acids, such as olive oil and fish oil, have been proven to have positive effects in reducing inflammation and improving cognitive function [5,6]. Moreover, recent studies have begun to focus on the benefits of specific functional factors present in foods for brain health. It has been reported that functional factors like vitamin D and vitamin E in food can effectively improve the anti-inflammatory and antioxidant status of the brain, thereby enhancing cognitive function [7]. These findings provide a scientific basis for developing novel nutritional interventions, such as incorporating lipid-soluble functional factors into edible oils, to target mechanisms of reducing brain inflammation and enhancing antioxidant defenses, thereby effectively alleviating the global burden of cognitive impairment.

Edible oils contain various components that are beneficial to brain cognitive function, including unsaturated fatty acids, vitamin E, and phospholipids [8]. Among these, unsaturated fatty acids can improve blood circulation, while vitamin E exhibits strong antioxidant properties that help protect neurons [9]. However, phospholipids, a key component of edible oils, are particularly noteworthy for their contribution to brain health. Phospholipids primarily include lecithin and cephalin, which play critical roles in the construction of neuronal cell membranes and signal transduction [10]. Studies have shown that phospholipids enhance cognitive function by improving cholinergic activity, thereby promoting the synthesis and release of acetylcholine [11]. Additionally, phospholipids possess antioxidant properties that help reduce oxidative stress in neurons, thereby protecting neural cells (Kidd, 1999). Notably, sunflower lecithin, which is rich in unsaturated fatty acids and antioxidants, shows significant potential in improving cognitive function. Phosphatidylcholine (PC) and phosphatidylserine (PS) found in sunflower lecithin have been demonstrated to support neuronal function and contribute positively to brain health [9].

Vitamin D plays a significant role in cognitive function. Research indicates that vitamin D can activate the PI3K-AKT signaling pathway, which promotes neuronal survival and synaptic plasticity [12,13]. By regulating the calcium ion balance, vitamin D fosters neuronal growth and enhances synaptic plasticity, thereby improving cognitive function [14]. Additionally, vitamin D possesses anti-inflammatory and antioxidant properties that protect neurons from damage [15]. Further studies have demonstrated that vitamin D increases the expression of key enzymes in the folate biosynthesis pathway, such as sepiapterin reductase (encoded by *Spr*) and dihydropteridine reductase (encoded by *Qdpr*). The upregulation of these enzymes enhances the synthesis of dopamine and serotonin (5-HT), which in turn improves attention and executive functions, thereby enhancing cognitive abilities in mice [16]. Furthermore, extensive literature reports that vitamin A, once metabolized into retinoic acid, can activate the retinoic acid signaling pathway through RAR/RXR receptors, facilitating neurodevelopment and improving synaptic plasticity [17]. In mouse models of inflammation, vitamin A has been shown to reduce inflammatory responses and decrease oxidative stress markers, such as malondialdehyde (MDA), by inhibiting nuclear factor kappa B (NF-κB). Such antioxidant and anti-inflammatory effects contribute to the protection of hippocampal neurons [18]. Multiple studies have established a strong association between deficiencies in vitamins D and A and declines in cognitive function. Supplementation with these vitamins has been shown to effectively improve cognitive impairments.

Given the important roles of vitamin D and vitamin A in enhancing cognitive function, this study explores the potential of incorporating these two vitamins into sunflower oil, which retains a high content of sunflower lecithin through specific processing techniques. This composite edible oil aims to provide nutritional supplementation of vitamins A and D while also exploiting the bioactive properties of sunflower lecithin. By targeting multiple pathways involved in brain health, this approach holds promise as a simple and effective solution for improving cognitive function, supporting healthy aging, and preventing cognitive disorders.

## 2. Material and Methods

### 2.1. Materials and Chemicals

The feed supplemented with vitamin A, vitamin D, and sunflower lecithin used in this study was purchased from Jiangsu Synergy Pharmaceutical Bioengineering Co., Ltd. (Nanjing, China). The sunflower oil used in the feed formulation was generously provided free of charge by Standard Foods Co., Ltd. (Taiwan, China). The chemical structures of Vitamin D and Vitamin A, as well as the composition of sunflower lecithin, can be found in the Supplementary Materials. Scopolamine hydrobromide (98% purity) was obtained from Shanghai Macklin Biochemical Co., Ltd. (Pudong, Shanghai, China). The assay kits for interleukin-6 (IL-6), glutathione (GSH), malondialdehyde (MDA), dopamine (DA), serotonin (5-HT), acetylcholine (ACh), and acetylcholine receptor (AChR) were sourced from Xiamen Huijia Biotechnology Co., Ltd. (Xiamen, China). Reverse transcription kits and universal high-sensitivity dye-based qPCR kits were provided by Nanjing Vazyme Biotech Co., Ltd. (Nanjing, China). Gene primers were synthesized by Shanghai Sangon Biotech Co., Ltd. (Shanghai, China). Chloral hydrate was purchased from Sinopharm Chemical Reagent Co., Ltd. (Beijing, China).

### 2.2. Animals

Male ICR mice (5 weeks old, 21 ± 2 g, specific pathogen free (SPF)) were purchased from Shanghai Slack Laboratory Animal Co., Ltd. (Shanghai, China). The animals used in this study and the animal experiments conducted were in compliance with the guidelines of the Declaration of Helsinki and were approved by the Animal Ethics Committee of Jiangnan University (JN. No20230615i1000906{304}). The mice were housed under a 12-h light/dark cycle, with free access to water and a custom diet, and were acclimated for one week before the experiments began. The experimental design was optimized to minimize the number of animals used and to reduce pain and distress in the animals.

### 2.3. Grouping and Intervention

Scopolamine, an anticholinergic drug, is widely used to induce memory deficits in mice by blocking acetylcholine’s action in the central nervous system. By inhibiting acetylcholine receptor activity, it mimics the pathological features of cognitive disorders, including memory decline and reduced learning ability [19]. Due to its ability to induce memory impairment rapidly and reversibly, scopolamine is commonly used to establish mouse models of cognitive dysfunction [20]. Such models are instrumental in studying the effects of various interventions on cognitive function and exploring potential therapeutic approaches. Therefore, this study utilized scopolamine to create a mouse model of memory impairment.

The experimental procedure is illustrated in the accompanying figure. After a one-week acclimation period, male ICR mice were randomly divided into the following six groups: the control group (Con), rivastigmine positive control group (Riv), scopolamine model group (Sop), vitamin D group (VD), vitamin A + vitamin D group (VD + VA), sunflower lecithin group (P), and vitamin A + sunflower lecithin group (P + VA), with ten mice in each group. The Con group received a daily intraperitoneal injection of normal saline, while the other groups were administered a daily intraperitoneal injection of a scopolamine solution (dissolved in sterile normal saline) at a volume of 10 mL/kg. Simultaneously, each group was fed a specific custom diet for intervention, with the diet formulation and energy composition provided in Table 1. The mice had free access to water and food, and their body weights were measured weekly. After four consecutive weeks of modeling and intervention, behavioral tests were conducted, including the open field test (OFT), novel object recognition test (NORT), Y-Maze test (YMT), and Morris water maze test (MWM). Following these tests, the mice were fasted for 12 h, then anesthetized with an intraperitoneal injection of 10% chloral hydrate, and subsequently euthanized by decapitation. Blood, cerebral cortex tissues, and hippocampus tissues were collected for further analysis.

### 2.4. Open Field Test

The open field test (OFT) is primarily used to evaluate spontaneous activity, exploratory behavior, and anxiety-like behavior in rodents. It provides valuable insights into an animal’s emotional state and general activity levels. The apparatus consists of a box measuring 40 cm in length, 40 cm in width, and 50 cm in height and is equipped with an infrared camera above to track a mouse’s movement. The floor of the testing area is divided into sixteen 10 × 10 cm^2^ areas, with the central four squares defined as the center zone. Each mouse is gently placed at the center point of the open field, and the researcher immediately leaves the apparatus to allow the mouse to move freely for 8 min. Video recording software is used to track the time the mouse spends in the center area. After each trial, any hair, urine, or feces left by the mouse is cleaned up. The apparatus is then wiped down with 75% ethanol to eliminate scent traces, which might affect the next mouse’s behavior. The ethanol is dried using tissues and a hairdryer before the next mouse is tested. The environment is kept consistent in terms of lighting and temperature to avoid confounding variables. Results are typically analyzed by measuring the time spent in the center zone and other exploratory behaviors.

### 2.5. Noval Object Recognition Test

The novel object recognition test (NORT) is a method used to evaluate learning and memory by leveraging the innate tendency of rodents to explore novel objects. The setup is similar to that used in the open field test. The experiment is divided into the following three phases: habituation, familiarization, and testing, with each phase separated by a 24-h interval. During the habituation phase, a mouse is placed in the experimental box to freely explore the environment for 8 min, which helps reduce its novelty and fear of the unfamiliar surroundings. In the familiarization phase, two identical cylindrical blocks (4 cm in diameter at the base; 10 cm in height) are placed on one side of the box, 5 cm from each corner. The mouse is placed in the center of the opposite side, facing away from the blocks, and allowed to explore the blocks and environment freely for 8 min. Twenty-four hours later, one of the cylindrical blocks is replaced with a conical block (4 cm in diameter at the base; 10 cm in height). The mouse is then reintroduced to the box using the same method as that during the familiarization phase and allowed to explore for another 8 min. An infrared camera records the time (T) that the mouse spends exploring both the novel and familiar objects. The box is wiped with 75% ethanol after each trial to eliminate any residual odors that might affect subsequent trials. The primary measure of interest is the discrimination index (DI), calculated using the formula: DI = (T_novel − T_familiar)/T_total exploration, which indicates the mouse’s ability to recognize and distinguish the new object from the familiar one.

### 2.6. Y-Maze Test

The Y-Maze test is primarily used to evaluate working memory or reference memory in rodents. The apparatus consists of three horizontal arms, each 70 cm in length and 15 cm in height, arranged at a 120° angle to each other. The arms are made of opaque polyethylene plastic. During the experiment, the arms are arbitrarily labeled as a, b, and c. At the start of the test, a mouse is placed in one of the arms, and an infrared camera tracks the sequence and number of entries into each arm. An entry sequence where the mouse consecutively enters different arms is defined as an alternation (e.g., abc, bac, or cba but not aca). The primary measure of the Y-Maze is spontaneous alternation, calculated as follows: spontaneous alternation = number of alternations/(total number of arm entries − 2). Each test session lasts for 5 min. After each trial, the maze is wiped with 75% ethanol to eliminate any residual odors that could influence subsequent tests.

### 2.7. Morris Water Maze Test

The Morris water maze test is one of the most classical methods for assessing spatial memory in rodents. The apparatus consists of a circular pool with a diameter of 120 cm and a height of 60 cm. The pool is divided into four quadrants labeled according to the cardinal directions—north, south, east, and west. Adjustable light sources are positioned on both sides to regulate the brightness of the water surface, and a camera is mounted above to record data. During the experiment, the pool is filled with 40 cm of water, dyed black with ink, and maintained at a temperature of 21 ± 1 °C. The experiment is divided into the following two phases: place navigation and a spatial probe. After each swim trial, care is taken to keep the mice warm by drying them with a clean towel or using a hairdryer. A platform is placed 1 cm below the water surface and remains fixed in one quadrant throughout the training. At the start of each trial, the mouse is placed in the pool facing the wall from one of the quadrants. An infrared camera tracks the mouse’s swimming path. The test duration is 60 s, and the time taken for the mouse to locate the platform is recorded as the escape latency. Upon finding the platform, the mouse is allowed to remain on it for 5 s. If the mouse fails to find the platform within the allotted time, the escape latency is recorded as 60 s. Mice that do not find the platform are guided to it and kept on it for 15 s. Each mouse undergoes four training trials daily, starting from different quadrants, with at least a 30-min interval between trials for the same mouse. This training continues for five consecutive days. It is important that during the guidance process, mice are not placed directly on the platform; instead, a guiding stick is used to direct the mouse’s swimming direction, encouraging the mouse to learn how to sense and climb onto the platform, thus remembering its location. On the sixth day, the spatial probe test is conducted by removing the platform from the pool. The mouse is placed into the pool facing the wall from the quadrant diagonally opposite to where the platform was previously located. The test duration is 60 s, and parameters such as swim speed, latency, distance traveled, and the number of times the mouse crosses the platform location are recorded. During the spatial probe phase, no scopolamine is administered.

### 2.8. Biochemical Index Detection

The measurement of biochemical indices in mice was based on previous experiments with some modifications. Blood was collected from the retro-orbital sinus and placed in an EP tube containing sodium heparin. The blood was then centrifuged at 3000 r/min for 15 min, and the supernatant was collected to measure IL-6, GSH, and MDA levels. After blood collection, the cerebral cortex and hippocampus were carefully extracted, quickly homogenized into a 10% tissue homogenate in pre-cooled sterile saline, and centrifuged at 12,000 r/min for 10 min at 4 °C. The supernatant was carefully collected for further analysis. Enzyme-linked immunosorbent assay (ELISA) kits, according to the manufacturer’s instructions (Xiamen Huijia Biotechnology Co., Ltd.) (Xiamen, China), were used to measure the respective biochemical markers. The supernatant from the cerebral cortex was used for IL-6 detection, while the hippocampal supernatant was used to determine the levels of dopamine (DA), serotonin (5-HT), acetylcholine (ACh), and acetylcholinesterase (AChE). The remaining samples were stored at −80 °C for future use.

### 2.9. Transcriptome Sequencing

Total RNA was isolated from the hippocampus using TRIzol reagent. RNA quality assessment, library construction, and transcriptome sequencing were performed by Shanghai Sangon Biotech Co., Ltd. (Shanghai, China). The raw sequencing data were first assessed for quality using FastQC to ensure its reliability. Subsequently, quality trimming was performed with Trimmomatic to remove low-quality reads, resulting in accurate and valid data for downstream analysis. Gene expression levels were evaluated using StringTie and leveraging known gene models to provide precise quantification of gene expression. Differential gene expression analysis was conducted using DESeq2, with selection criteria set at a *p*-value < 0.05 and a fold change (FC) greater than 1.5. Visualization and clustering analysis were carried out based on the results of the differential analysis. Kyoto Encyclopedia of Genes and Genomes (KEGG) pathway enrichment analysis was conducted using the clusterProfiler package (3.0.5), and Gene Ontology (GO) enrichment analysis was performed using TopGO [21].

### 2.10. Gene Expression in the Hippocampus Was Detected by qPCR

Total RNA was extracted from the hippocampus using the TRIzol method. The purity of the extracted RNA was assessed by measuring the A260 nm/A280 nm ratio, and a ratio between 1.8 and 2.0 indicated acceptable purity. Reverse transcription was carried out following the instructions of the reverse transcription kit. The 20 μL reaction system consisted of 1 μg of total RNA, 4.0 μL of 5× All-in-one qRT SuperMix, 1.0 μL of Enzyme Mix, and RNase-free dH2O to a final volume of 20 μL. The reverse transcription conditions were set as follows: 50 °C for 15 min, followed by 85 °C for 5 s. The resulting cDNA was stored at −80 °C for future use [22]. Quantitative detection of gene expression was performed using the SYBR Green qPCR kit, with β-actin as the internal reference gene. The primer sequences are provided in Table 2. The qPCR reaction system (10 μL total volume) consisted of 0.2 μL of forward primer (10 μmol/L), 0.2 μL of reverse primer (10 μmol/L), 5.0 μL of 2× ChamQ Universal SYBR qPCR Master Mix, 3.6 μL of sterile water, and 2 μL of cDNA template. The Ct values for each template were determined using a qPCR instrument, and relative quantification was calculated using the 2^−ΔΔCt^ method [23].

### 2.11. Data Analysis

The experimental data were analyzed using IBM SPSS Statistics 27.0 software, and graphs were created using Origin 2022 software. After confirming that the data met the assumptions of a normal distribution and homogeneity of variance, one-way analysis of variance (ANOVA) was conducted. Tukey’s S-B (Studentized Range) test was used to determine the statistical significance between different treatment groups when significant differences were identified among groups. For data with non-homogeneous variances, Tamhane’s T2 test was applied [22]. A *p*-value less than 0.05 was considered indicative of statistically significant differences between groups.

## 3. Results

### 3.1. Growth Performance and Feed Intake

As shown in Table 3, five weight measurements were conducted during the modeling and intervention period, each spaced one week apart. It was observed that, except for the rivastigmine positive control group (Riv group), there were no significant differences in body weight among the groups of mice. The Riv group showed a trend of weight reduction during the second and third weeks, which might be related to the method and concentration of the agent administered. There were no significant differences in food intake among the different groups of mice.

### 3.2. The Added Functional Factors Improved the Spontaneous Activity Behavior and Exploration Behavior of Mice

**Figure 1:**
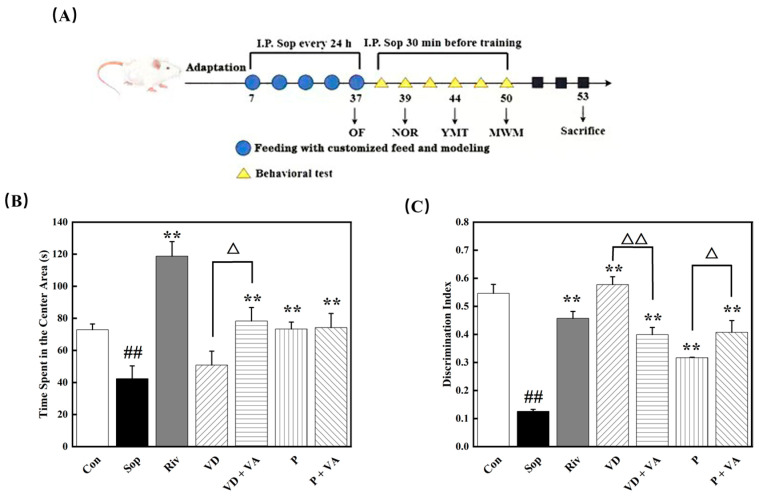

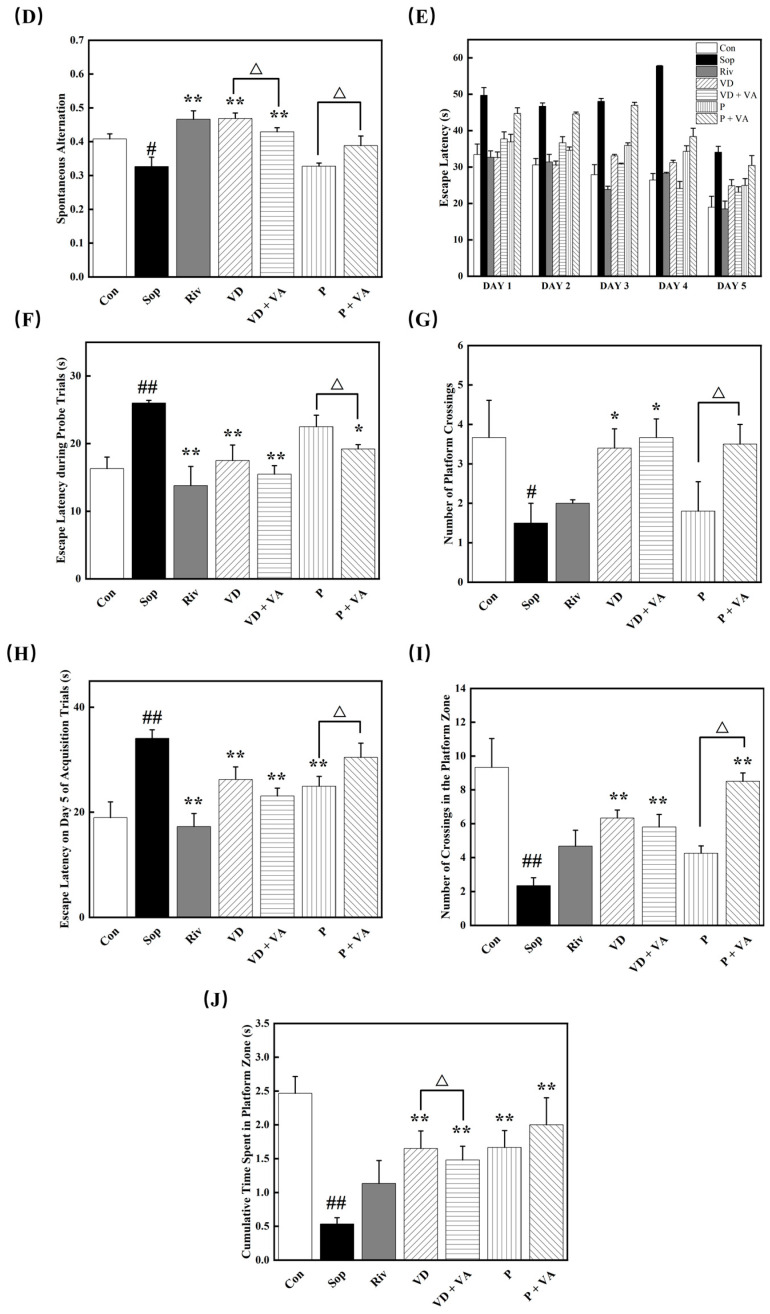
Sunflower lecithin, vitamin D, and vitamin A added to sunflower oil improved the behavioral indices of mice. (**A**) Schematic diagram of the procedures for animal experiments. (**B**) Cumulative time spent by mice in the center area of the open field. (**C**) Discrimination index of mice in the novel object recognition test. (**D**) Discrimination index of mice in the Y-Maze test. (**E**) Statistical comparison of the five-day escape latency of mice during the acquisition phase. (**F**) Comparison of the escape latency of the mice on day five of the acquisition phase. (**G**) The number of platform crossings by mice during the acquisition phase. (**H**) Escape latency of the mice during the probe trial. (**I**) Cumulative time spent by mice in the platform zone during the probe trial. (**J**) The number of platform zone crossings by mice during the probe trial. All values are presented as the mean ± SD. * *p* < 0.05, ** *p* < 0.01 versus the Sop group; # *p* < 0.05, ## *p* < 0.01 versus the Con group; △ *p* < 0.05, △△ *p* < 0.01, comparing the VD group with the VD + VA group or the P group with the P + VA group, *n* = 10.

The open field test (OFT) was used to investigate the effects of supplementation with sunflower lecithin, vitamin D, and vitamin A on exploratory behavior in scopolamine-treated mice. In a novel environment, the exploratory behavior of mice can be assessed by the amount of time they spend in the center area. A longer duration in the center area indicates a greater willingness to explore open spaces, reflecting higher exploratory motivation and lower anxiety levels [24]. As shown in Figure 1B, compared to the control group, the scopolamine model group exhibited a significant reduction in the time spent in the center area (*p* < 0.05). Supplementing the diet with sunflower lecithin, vitamin D, and vitamin A effectively increased the exploratory behavior of the mice. In the model group, the average time spent in the central area was 42.34 s. In contrast, the mice in the VD + VA, P, and P + VA groups exhibited significantly prolonged times in the central area, with an average duration exceeding 70 s, representing a 1.6-fold increase. Notably, the sunflower lecithin group and the combination groups demonstrated particularly strong improvements.

### 3.3. Intervention with Functional Factors and Effects on Recognition Disorders in Mice

The novel object recognition (NOR) test was employed to explore the effects of functional factors on overcoming object recognition impairment in mice. In the NOR test, mice are exposed to two objects, including one familiar object and one novel object. Healthy mice typically spend more time exploring the novel object, driven by their interest and curiosity. If mice can recognize the novel object and spend more time engaging with it, this indicates that their memory function is intact. Thus, the discrimination index effectively reflects the memory function of the mice [25]. As shown in Figure 1C, the discrimination index of the model group was significantly lower than that of the control group (*p* < 0.05). The discrimination index in the model group had an average value of 0.126, whereas the VD group exhibited a mean discrimination index of 0.578, indicating a 4.5-fold increase. This suggests that vitamin D had a particularly strong effect in helping mice overcome novel object recognition impairment. The other intervention groups also showed significant improvements in the discrimination index. Moreover, the combination of P and VA further enhanced the mice’s ability to recognize novel objects compared to P alone.

### 3.4. Improving the Effects of Functional Factors on Working Memory in Mice

The Y-Maze test (YMT) was used to evaluate the effects of vitamin A and combination regimens on working memory in mice. Spontaneous alternating behavior in this test reflects the working memory capacity of mice. Working memory involves the ability to retain and manipulate information over short periods. In the Y-Maze test, mice tend to explore a new arm rather than returning to the one they have recently visited, which requires them to remember where they have been. Successful spontaneous alternation indicates good working memory [26]. As shown in Figure 1D, the number of spontaneous alternations in the scopolamine model group was significantly lower than that in the control group (*p* < 0.05). Except for the sunflower lecithin group, all other intervention groups showed a significantly increased number of spontaneous alternations. Among the interventions, vitamin D showed a particularly strong effect on improving working memory.

### 3.5. Improving the Effects of Functional Factors on Spatial Memory in Mice

The Morris water maze test (MWM) was employed to assess the effects of vitamin A and its combination with other supplements on the spatial memory of mice. By allowing the mice to search for a hidden platform in the pool, their ability to remember and utilize environmental cues was evaluated, making it particularly suitable for studying spatial memory impairments, such as those induced by scopolamine. As shown in Figure 1E, during the acquisition phase, the escape latency gradually decreased with the increasing number of training days, indicating effective training. On the fifth day (Figure 1F), the escape latency of the model group was significantly longer than that of the control group, indicating a marked difference. The intake of VD, P, and VA significantly shortened the escape latency in mice, with the combination of sunflower lecithin and vitamin A showing the best results. Additionally, both the vitamin D group and the combination groups showed significantly increased numbers of platform crossings (Figure 1G) during the acquisition phase, while the effect of sunflower lecithin alone was less pronounced. During the probe trial, where the platform was removed, the escape latency of the model group was significantly longer than that of the control group (*p* < 0.05). Except for the VD + VA group, all intervention groups demonstrated significantly shortened escape latency (Figure 1H). Moreover, the cumulative time spent in the platform zone (Figure 1I) and the number of platform zone crossings (Figure 1J) in the intervention groups were significantly higher than those in the model group, indicating that the mice retained a good memory of the platform location and thus demonstrating strong spatial memory abilities. Overall, the improvement in spatial memory was more pronounced in the groups receiving vitamin D alone and in the group with combined supplementation of sunflower lecithin, vitamin D, and vitamin A.

### 3.6. Functional Factors Added to Sunflower Oil Alleviated Scopolamine Induced Oxidative Stress

**Figure 2:**
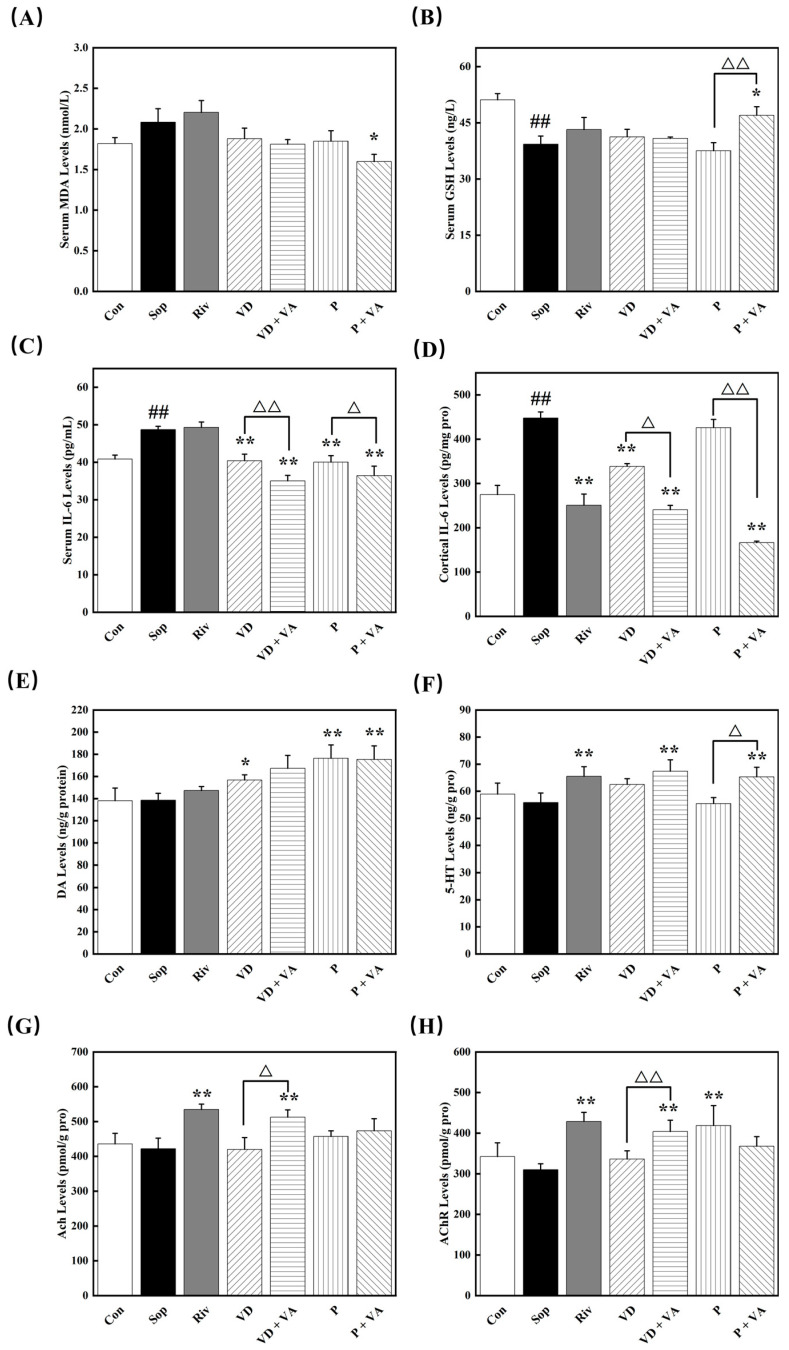
The addition of sunflower lecithin, vitamin D, and vitamin A to sunflower oil reduces oxidative stress and inflammation levels and increases monoamine neurotransmitter contents in the hippocampus of mice. (**A**) Serum malondialdehyde (MDA) levels. (**B**) Serum glutathione (GSH) levels. (**C**) Serum interleukin-6 (IL-6) levels. (**D**) Cortical interleukin-6 (IL-6) levels. (**E**) Dopamine (DA) levels. (**F**) Serotonin (5-HT) levels. (**G**) Acetylcholine (ACh) levels. (**H**) Acetylcholine receptor (AChR) levels. All values are presented as the mean ± SD. * *p* < 0.05, ** *p* < 0.01, versus the Sop group; ## *p* < 0.01 versus the Con group; △ *p* < 0.05, △△; *p* < 0.01, comparing the VD group with the VD + VA group or the P group with the P + VA group, *n* = 10.

Numerous studies have shown that oxidative stress is one of the key pathogenic mechanisms in neurodegenerative diseases. The body mitigates oxidative stress by upregulating antioxidant enzyme activity and utilizing endogenous natural antioxidants to neutralize excess reactive oxygen species (ROS). Currently, several indicators reflect the oxidative status of the body, such as glutathione (GSH) and malondialdehyde (MDA). In this study, we measured these two blood indicators to evaluate the effects of Sunflower lecithin, vitamin D, and their combination on the antioxidant capacity of mice. Accumulation of MDA can cause cross-linking and polymerization of biomolecules, including nucleic acids and proteins, leading to cytotoxic effects. Therefore, the MDA content is a crucial indicator of the extent of oxidative damage in the body. Figure 2A indicates that MDA levels were higher in the Sop group than in the Con group, although this difference did not reach statistical significance. The P+VA group showed significantly reduced MDA contents in the blood (*p* < 0.05). Regarding GSH levels (Figure 2B), the P + VA group showed effectively increased serum GSH contents (*p* < 0.05), while the other intervention groups showed a trend of improvement but without statistical significance.

### 3.7. Functional Factors Added to Sunflower Oil Reduce Scopolamine-Induced Inflammation

Studies have shown that cognitive impairment and memory dysfunction are influenced not only by oxidative stress in the brain but also by inflammatory responses that can damage neuronal cells and synaptic structures. As shown in Figure 2D, the VD, VD + VA, and P + VA groups had significantly reduced IL-6 levels in the cerebral cortex. In the model group, the IL-6 level was 448 pg/mg protein, whereas the P + VA group exhibited an IL-6 level of 166 pg/mg protein, a reduction of 2.6-fold compared to that in the model group. There was no significant difference between the P-alone group and the model group. All four intervention groups had significantly lowered serum IL-6 levels (Figure 2C), with the combination groups showing more pronounced effects. These results indicate that combining vitamin D and sunflower lecithin with vitamin A can further improve neuronal inflammation levels in the brains of mice.

### 3.8. Functional Factors Added to Sunflower Oil Can Improve Mouse Cognition by Increasing the Content of Monoamine Neurotransmitters

Acetylcholine (ACh) is a key neurotransmitter involved in various brain functions, particularly learning and memory. ACh promotes neuronal survival and synaptic plasticity by binding to its receptors, such as the M1 muscarinic receptor, and activating multiple signaling pathways. Serotonin (5-HT) plays a crucial role in regulating mood, cognition, and memory. Studies have shown that increasing 5-HT levels can ameliorate cognitive impairment induced by scopolamine. Dopamine (DA) is another important neurotransmitter associated with reward and motivation. Increased dopamine levels in the hippocampus contribute to enhanced learning and memory functions. As shown in Figure 2G,H, the sunflower lecithin group showed significantly increased levels of ACh and AChR in the hippocampus of mice. The combination groups showed significant effects on the synthesis of 5-HT (Figure 2F) and DA (Figure 2E). These findings suggest that supplementation with vitamin D and sunflower lecithin, in combination with vitamin A, further enhances monoamine neurotransmitter levels in the mouse hippocampus.

### 3.9. Transcriptome Analysis Revealed the Mechanism of Sunflower Lecithin, Vitamin D. and Their Combination in Improving Learning and Memory in Mice

**Figure 3:**
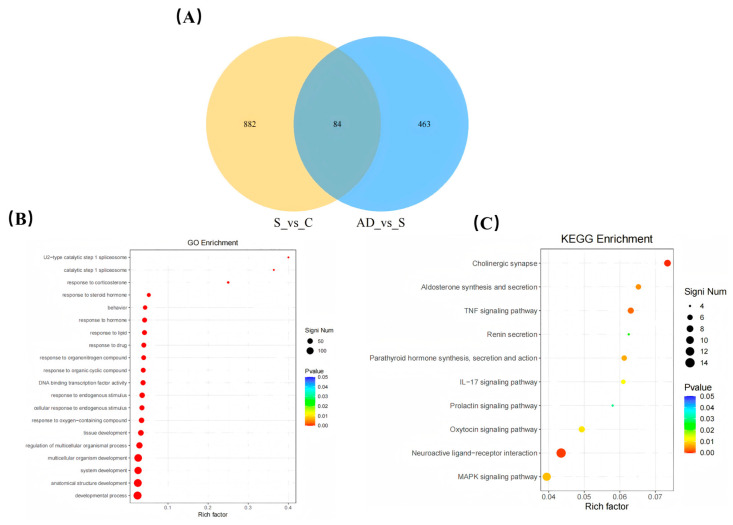
Transcriptomic sequencing results of the hippocampus in the VD + VA group. (**A**) Venn diagram showing the overlap between the Sop/Con and VD + VA/Sop comparisons, illustrating the common and unique DEGs. (**B**) Gene ontology (GO) functional enrichment bubble plot of all DEGs in the VD + VA group, with the Sop model group as the control. GO terms enriched in the DEGs are represented as bubbles, with the size indicating the number of genes involved and the color reflecting the significance level. (**C**) KEGG pathway enrichment bubble plot of all DEGs in the intervention group, with the Sop model group as the control. KEGG pathways enriched in the DEGs are represented as bubbles, with the size indicating the number of genes involved and the color reflecting the significance level. All data are presented as the mean ± SEM, *n* = 3.

To elucidate the potential molecular mechanisms by which sunflower lecithin, vitamin D, and their combination ameliorate scopolamine-induced cognitive impairment, we conducted an RNA-seq analysis on the control group, the model group, and the four intervention groups. A heatmap of differentially expressed genes (DEGs) between the intervention groups and the model group was generated based on log2 (FC) values, with the number of differentially expressed genes listed in Table 4. Taking the VD + VA group as an example, vitamin D supplementation effectively modulated the expression of 84 genes previously altered by scopolamine, either upregulating or downregulating them (Figure 3A). The GO functional enrichment bubble plot indicates that the VD + VA group had significant differences from the model group in terms of “Behavior”, “Response to steroid hormone”, “Response to hormone” and “Response to organic cyclic compound”. “Behavior” is directly related to cognitive and behavioral functions, making it central to studies on cognitive impairment. “Response to steroid hormone” and “Response to hormone” are closely linked to the improvement of cognitive disorders, as steroid hormones play a crucial role in brain function and may be involved in cognitive processes. “Response to organic cyclic compound” includes important neurochemicals and molecules related to brain function, which are essential for cognitive health (Figure 3B). According to the KEGG classification results, the VD + VA treatment primarily exerted its effects through pathways such as cholinergic synapses, neuroactive ligand–receptor interactions, and the TNF signaling pathway (Figure 3C). This suggests that the VD+VA intervention enhances neuronal synaptic plasticity and reduces inflammation by modulating cholinergic pathways and neuroactive ligand–receptor interactions, thereby offering neuroprotection and alleviating neuroinflammation. Based on the KEGG pathway analysis of each intervention group, we selected the PI3K-AKT signaling pathway, cholinergic synapses, and folate biosynthesis as the focus of our study. Key genes within these pathways were validated using qPCR.

### 3.10. Mechanism of Synergistic Improvement in Learning and Memory in Mice by Vitamin D, Sunflower Phospholipid, and the Combination Intervention

**Figure 4:**
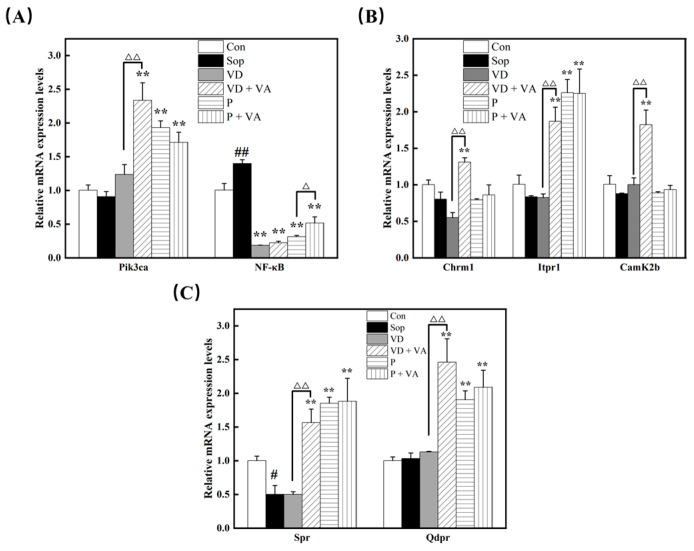
Addition of sunflower lecithin, vitamin D, and vitamin A in sunflower oil improves cognitive impairment in mice via the PI3K-AKT signaling pathway, cholinergic signaling pathway, and folate biosynthesis. (**A**) Relative levels of *Pik3ca* and *NF-κB* in the hippocampus. (**B**) Relative levels of *Chrm1*, *Itpr1*, and *Camk2b* in the hippocampus. (**C**) Relative levels of *Spr* and *Qdpr* in the hippocampus. All values are presented as mean ± SD. ** *p* < 0.01, versus Sop group; # *p* < 0.05, ## *p* < 0.01 versus Con group; △ *p* < 0.05, △△ *p* < 0.01, comparing the VD group with the VD + VA group or the P group with the P + VA group, *n* = 3.

To understand how the intervention groups developed alleviated scopolamine-induced cognitive impairment, we examined the expression levels of genes associated with the PI3K-AKT signaling, cholinergic signaling, and folate biosynthesis pathways in the hippocampus. As depicted in Figure 4A, all four intervention groups showed significantly upregulated *Pik3ca* (encoding PI3K) and significantly downregulated *NF-κB*. This suggests that the addition of vitamin D and sunflower lecithin reduces inflammatory responses in the mouse hippocampus through the PI3K-AKT signaling pathway, thereby decreasing inflammation-mediated neuronal apoptosis and synaptic damage. This regulatory mechanism helps protect neuronal structures in the hippocampus, maintain synaptic function, and enhance long-term potentiation (LTP) and synaptic plasticity.

Within the cholinergic pathway (Figure 4B), the VD + VA intervention significantly increased the expression of Chrm1, the gene encoding the muscarinic acetylcholine receptor M1, which is crucial for neurotransmission and synaptic plasticity. Additionally, the increased expression of Itpr1 (encoding IP3R) and Camk2b (encoding calcium/calmodulin-dependent protein kinase IIβ) suggests that the VD+VA intervention further activates Gq/11 protein-coupled signaling pathways via the cholinergic pathway. These findings suggest that the VD+VA treatment has a significant impact on the cholinergic signaling components involved in neurotransmission and synaptic plasticity.

Regarding the folate biosynthesis pathway, except for the VD intervention, the other three interventions significantly upregulated Spr and Qdpr, which encode sepiapterin reductase and dihydropteridine reductase, respectively (Figure 4C). This suggests that these interventions improve the synthesis of tetrahydrobiopterin (BH4), a cofactor involved in neurotransmitter synthesis. The levels of dopamine and serotonin, as well as redox indicators, in mouse blood were consistent with the qPCR results.

## 4. Discussion

This study demonstrated that the combination of vitamin A, vitamin D, and sunflower lecithin alleviates scopolamine-induced cognitive impairment through the regulation of key signaling pathways, such as the PI3K-AKT and cholinergic pathways, and by modulating neurotransmitter levels (dopamine and serotonin) and synaptic plasticity. These interventions also help reduce inflammation and oxidative stress, thereby improving cognitive function in mice. The combined use of vitamin D and sunflower lecithin with vitamin A showed superior intervention effects.

Sunflower oil, rich in unsaturated fatty acids, and its derived sunflower lecithin have been shown to offer numerous health benefits, particularly in enhancing brain cognition and function. The metabolism of sunflower lecithin within the body involves the breakdown of its phospholipids into choline, which is a critical precursor for acetylcholine, a neurotransmitter that is essential for cognitive processes [27]. Studies have demonstrated that supplementation with sunflower oil, due to its high content of omega-6 fatty acids, can influence the lipid composition of neuronal membranes, potentially enhancing synaptic plasticity and improving memory function [28]. Furthermore, sunflower lecithin’s antioxidative effects may contribute to neuroprotection. Rich in phospholipids like phosphatidylcholine, sunflower lecithin plays a key role in maintaining the structure and integrity of biological membranes. As observed in our results, it influences cellular antioxidant defense, helping to maintain membrane fluidity and modulate membrane-associated enzymes. This regulation supports the antioxidant defense system. In our study, we noted a significant decrease in MDA levels and an increase in GSH contents in the treatment group, indicating an enhancement of cells’ antioxidant capacity. This effect is likely due to sunflower lecithin’s ability to promote GSH regeneration and reduce lipid peroxidation. Additionally, by lowering lipid peroxidation products like MDA, sunflower lecithin may mitigate oxidative stress-induced damage to cellular structures, including nucleic acids and proteins. These findings underscore the dual role of sunflower lecithin, not only in supplying essential fatty acids but also in supporting cognitive function through its antioxidative properties [28]. These findings underline the dual role of sunflower lecithin in both supplying essential fatty acids and supporting cognitive function through its antioxidative properties.

Both vitamin D and vitamin A are essential for cognitive health, with vitamin D promoting neuroprotection through PI3K-AKT signaling and vitamin A supporting neurogenesis and synaptic plasticity. Once ingested, vitamin D undergoes hydroxylation in the liver to form 25-hydroxyvitamin D, which is subsequently converted in the kidneys to its active form, 1,25-dihydroxyvitamin D (calcitriol). This active form binds to vitamin D receptors (VDRs) in the brain, regulating gene expression related to neuroprotection, reducing neuroinflammation, and supporting synaptic plasticity, all of which are vital for cognitive health [29]. Vitamin A, on the other hand, is metabolized into retinoic acid, which interacts with nuclear retinoic acid receptors (RARs) to modulate gene expression involved in neurogenesis, neurotransmitter synthesis, and synaptic function [30]. The combination of these vitamins with sunflower lecithin improves their bioavailability and stability, optimizing their effects on cognitive health. The antioxidant properties of vitamin A, combined with the neuroprotective effects of vitamin D, make sunflower oil an effective delivery medium for these functional agents to improve cognitive function [31]. The synergistic effects of these vitamins, facilitated by the oil’s unsaturated fatty acid content, underscore their potential in dietary strategies aimed at cognitive enhancement.

In both behavioral assessments and epigenetic markers, we observed that the combination of sunflower lecithin with vitamin A outperformed the sunflower lecithin-only intervention across the majority of parameters. Transcriptome sequencing analysis further indicated that supplementing sunflower lecithin with vitamin A significantly modulates key signaling pathways, including the mTOR signaling pathway, GABAergic synapses, cholinergic synapses, and the phosphatidylinositol signaling system. This combination resulted in the upregulation of a total of 60 associated genes within these pathways. The mTOR signaling pathway and GABAergic synapses play foundational roles in cognitive function by regulating synaptic plasticity and maintaining the excitatory–inhibitory balance, respectively. However, cholinergic synapses and the phosphatidylinositol signaling system are particularly critical for enhancing cognitive abilities given their more direct involvement in neurotransmitter release, synaptic plasticity, and cellular resilience. Cholinergic synapses, primarily through the action of acetylcholine, significantly modulate cognitive processes such as learning, memory, and attention. Acetylcholine activates muscarinic and nicotinic receptors, leading to the modulation of various intracellular signaling pathways that facilitate synaptic plasticity and the encoding of new information [32]. This cholinergic signaling is crucial for maintaining cognitive flexibility and has been shown to enhance long-term potentiation (LTP), a key mechanism underlying learning and memory [33]. The phosphatidylinositol signaling system plays a multifaceted role in cognition by regulating intracellular calcium levels and signaling cascades crucial for synaptic function. It generates second messengers such as inositol trisphosphate (IP3) and diacylglycerol (DAG), which are involved in calcium release from the endoplasmic reticulum. This calcium signaling is essential for neurotransmitter release and the induction of LTP, thereby supporting the structural and functional changes required for memory formation [34]. Furthermore, the PI3K/Akt branch of the phosphatidylinositol pathway is vital for neuronal survival, promoting cell growth and differentiation, which is important for maintaining synaptic health and cognitive function [35]. This pathway also contributes to neuroprotection by activating downstream effectors that reduce oxidative stress and inflammation, thus protecting neurons from damage and dysfunction [36]. Reducing inflammation and oxidative stress is a critical aspect of cognitive health, as chronic inflammation and oxidative damage are associated with cognitive decline and neurodegenerative diseases. The cholinergic system has anti-inflammatory properties, with acetylcholine interacting with the alpha-7 nicotinic acetylcholine receptor (α7nAChR) to suppress pro-inflammatory cytokine production [37]. Similarly, the phosphatidylinositol signaling system modulates oxidative stress responses by enhancing the activity of antioxidant enzymes and reducing the production of reactive oxygen species (ROS), thereby protecting neural tissue from oxidative damage [38]. In summary, while mTOR signaling and GABAergic synapses provide critical support for synaptic function and balance, the cholinergic synapse and phosphatidylinositol signaling system directly enhance cognitive function through their roles in neurotransmitter release, synaptic plasticity, and the regulation of inflammation and oxidative stress. These pathways work in concert to maintain neuronal health, enhance learning and memory, and protect against cognitive decline.

Experimental results have demonstrated that the combination of vitamin D and vitamin A significantly upregulates the expression levels of Chrm1, Itpr1, and Camk2b genes, which are pivotal for cholinergic signaling and cognitive function. This finding suggests a synergistic effect of these vitamins in enhancing the cholinergic pathway, which is crucial for learning, memory, and overall cognitive health. The Chrm1 gene encodes the muscarinic acetylcholine receptor M1, a receptor that plays a central role in modulating synaptic plasticity and neuronal excitability, processes that are essential for cognitive functions [39]. The upregulation of Chrm1 suggests that the combined action of vitamin D and vitamin A may enhance the sensitivity of neurons to acetylcholine, thereby improving cholinergic neurotransmission. Furthermore, the Itpr1 gene, which encodes inositol 1,4,5-trisphosphate receptor type 1, is a key regulator of intracellular calcium release. Calcium release is critical for various neuronal functions, including neurotransmitter release and activation of calcium-dependent signaling pathways. The increased expression of Itpr1 indicates that the vitamin D and vitamin A combination may potentiate calcium signaling, enhancing synaptic plasticity and memory formation [34]. The Camk2b gene encodes the calcium/calmodulin-dependent protein kinase II beta, a vital enzyme in the signaling cascades that lead to synaptic strengthening and long-term potentiation (LTP), both of which are central to the processes of learning and memory [40]. The synergistic effect of vitamin D and vitamin A on these pathways can be attributed to their combined influence on intracellular signaling mechanisms. Vitamin D, through its active metabolite 1,25-dihydroxyvitamin D, binds to vitamin D receptors (VDRs), which may interact with retinoic acid receptors (RARs) activated by vitamin A. This interaction can amplify the transcriptional activity of genes involved in calcium signaling and cholinergic function, such as Chrm1, Itpr1, and Camk2b. The activation of VDRs and RARs by these vitamins can lead to enhanced calcium influx, increased production of acetylcholine, and upregulation of acetylcholine receptors, thereby facilitating synaptic plasticity and cognitive enhancement [41]. Moreover, the co-administration of vitamin D and vitamin A not only boosts cholinergic signaling but also contributes to neuroprotection by reducing oxidative stress and inflammation. These vitamins can modulate the expression of antioxidant enzymes and anti-inflammatory cytokines, thus providing a protective environment that supports cognitive health [37,38]. By improving the cellular environment and enhancing cholinergic transmission, the combination of vitamin D and vitamin A offers a multifaceted approach to enhancing cognitive functions. Additionally, the combination of sunflower lecithin, vitamin D, and vitamin A significantly upregulated the expression of the Spr (encoding sepiapterin reductase) and Qdpr (encoding dihydropteridine reductase) genes, thereby increasing the synthesis of dopamine and 5-HT. The increased levels of these monoamine neurotransmitters not only strengthen the cholinergic pathway’s role in synaptic transmission between neurons but also support dopamine and 5-HT production through the folate biosynthesis pathway, improving reward mechanisms and motor behavior, regulating mood, and enhancing memory consolidation. Therefore, the synergistic action of vitamin D and sunflower lecithin with vitamin A, involving both cholinergic and folate biosynthesis pathways, comprehensively improved cognitive dysfunction in mice and positively impacted mood regulation and memory consolidation.

The intervention group led by the combination of vitamin D and vitamin A effectively improved cognitive memory impairment in mice and elucidated the potential underlying mechanisms. Upon binding of acetylcholine to *Chrm1*, the Gq/11 protein-coupled signaling cascade is first activated, leading to the production of IP3 and DAG mediated by phospholipase C (PLC). IP3 promotes calcium release from the endoplasmic reticulum via IP3R3 (encoded by *Itpr1*), enhancing intracellular calcium signaling, which in turn activates calcium/calmodulin-dependent protein kinase IIβ (encoded by *Camk2b*). Meanwhile, DAG, in conjunction with PKC, activates PI3K (encoded by *Pik3ca*), thereby initiating the downstream AKT signaling pathway. The activation of this pathway not only promotes the nuclear translocation of NF-κB, regulating gene expression related to cell survival and inflammatory responses but also directly enhances the maintenance of long-term potentiation (LTP) through the phosphorylation of CaMKIIβ. LTP is a core mechanism of synaptic plasticity, and its enhancement is crucial for improving cognitive functions. In summary, the results of this study indicate that the combined action of vitamin D and vitamin A significantly enhances the functionality of the cholinergic pathway by synergistically regulating the expression of *Chrm1*, *Pik3ca*, *Itpr1*, and *Camk2b*, thereby improving neuronal synaptic plasticity and cognitive abilities in mice. Additionally, the intervention groups showed increased gene expression levels of key enzymes such as sepiapterin reductase (encoded by *Spr*) and dihydropteridine reductase (encoded by *Qdpr*) in the folate biosynthesis pathway. This suggests that the three added functional factors improve the synthesis of BH4, thereby elevating monoamine neurotransmitter levels (such as dopamine and 5-HT) in the hippocampus, which in turn enhances attention, executive function, mood regulation, and learning and memory in mice. Moreover, BH4, a product of 7,8-dihydrobiopterin reductase, is involved in intracellular redox reactions, maintaining redox balance, indicating its benefits in helping hippocampal neurons resist oxidative stress. Overall, supplementation with Sunflower lecithin, vitamin D, and vitamin A in sunflower oil enhances hippocampal inflammation regulation, boosts monoamine neurotransmitter levels, and reduces oxidative stress and apoptotic markers, leading to improved cognitive functions in mice. Despite these promising findings, the exact molecular mechanisms responsible for the synergistic effects of vitamin D, vitamin A, and sunflower lecithin, as well as the optimal dosages for their combined use, require further investigation. Future studies using techniques such as siRNA or specific inhibitors are needed to clarify the roles of PI3K-AKT and cholinergic pathways in the effects of these interventions on memory impairment. Further studies in this area are warranted.

## 5. Conclusions

In conclusion, sunflower oil enriched with sunflower lecithin, vitamin D, and vitamin A significantly alleviated scopolamine-induced cognitive dysfunction in mice. The P + VA group exhibited stronger improvements in cognitive function, with reduced oxidative stress and inflammation and enhanced memory and learning abilities through the PI3K-AKT signaling pathway and cholinergic neurotransmission. Meanwhile, the VD + VA group also experienced significant benefits, primarily by modulating cholinergic synaptic activity and calcium signaling pathways. Both combinations demonstrated protective effects against cognitive impairment, with the P + VA group showing slightly superior efficacy in overall antioxidant and anti-inflammatory outcomes. These findings collectively demonstrate that the functional components—sunflower lecithin, vitamin D, and vitamin A—added to sunflower oil show potential in improving learning and memory impairments, thus playing a crucial role in the prevention of cognitive disorders.

## Figures and Tables

**Table 1:** Formulations and energy compositions of the diets.

Composition (g/kg)	Con	VD	VD + VA	P	P + VA
Casein	140	140	140	140	140
L-Cystine	1.8	1.8	1.8	1.8	1.8
Corn Starch	495.7	495.7	495.7	495.7	495.7
Maltodextrin 10	125	125	125	125	125
Sucrose	100	100	100	100	100
Cellulose	50	50	50	50	50
Sunflower Oil	40	40	40	40	40
Mineral Mix S10022M	35	35	35	35	35
Sunflower Lecithin	/	/	/	0.004	0.004
Vitamin Mix V10037	10	10	10	10	10
Vitamin E	86 mg	86 mg	86 mg	86 mg	86 mg
Vitamin D	/	23.2 µg	23.2 µg	/	/
Vitamin A	/	/	2363 µg	2363 µg	2363 µg
Choline Bitartrate	2.5	2.5	2.5	2.5	2.5
Total	1000	1000	1000	1000.004	1000.004
Protein/%	14.73	14.73	14.73	14.73	14.73
Carbohydrate/%	75.92	75.92	75.92	75.92	75.92
Fat/%	9.35	9.35	9.35	9.35	9.35

Note: The feed ingredients for the Con group and Sop group were the same, and only the feed ingredients for the Con group are listed in the table. Mineral Mix S10022M: a commercially available mineral supplement designed for use in laboratory animal diets, which was sourced from Jiangsu Synergy Pharmaceutical Bioengineering Co., Ltd. to ensure the appropriate mineral balance in the experimental diets for the study; Vitamin Mix V10037: a commercial supplement used to provide vitamins E, D, and A, as mentioned in the accompanying lines; the amounts of vitamin E, vitamin D, and vitamin A are the total contents of the vitamin mixtures.

**Table 2:** Real-time quantitative polymerase-chain-reaction primers.

Gene	Upstream Primer (5′−3′)	Downstream Primer (3′−5′)
*β-actin*	GGCTGTA TTCCCTCCA TCG	CCAGTTGGTAACAA TGCCA TGT
*Pik3ca*	CTGCAGTTCAACAGCCACAC	ATGCTGCTTGATGGTGTGGA
*NF-κB*	A TGGCAGACGA TGA TCCCTAC	TGTTGACAGTGGTA TTTCTGGTG
*Camk2b*	TGATGTCCTGAGCTTGGTGAG	GGGGGCTAATGGGAACTGG
*Spr*	GTAAGCGCACGCAGTGAGT	TCATTCACGTTGAGGAAGCCT
*Qdpr*	GTGCCAGCGTGGTTGTTAAG	GCTTCCACATCATGTCACAGTT
*Chrm1*	CTGGCAATACCTAGTTGGGGA	CCAGTACAGCGTACACATGAC
*Itpr1*	CGTTTTGAGTTTGAAGGCGTTT	CATCTTGCGCCAATTCCCG

**Table 3:** Growth performance and feed intake statistics of the mice in each group.

Group	Day 7 (g/piece)	Day 14 (g/piece)	Day 21 (g/piece)	Day 28 (g/piece)	Day 35 (g/piece)	Food Intake
Con	32.48	34.81	35.19	36.25	37.35	3.24 ± 0.12
Sop	33.39	34.67	34.88	36.02	37.00	3.03 ± 0.13
Riv	34.02	33.02	33.00	34.29	36.10	2.98 ± 0.15
VD	33.76	35.09	35.48	36.47	37.99	3.22 ± 0.17
VD + VA	33.68	34.18	35.05	36.18	37.47	3.23 ± 0.06
P	33.11	34.53	35.05	36.59	37.83	3.17 ± 0.2
P + VA	32.86	33.88	34.56	36.09	37.19	3.04 ± 0.19

Con: normal control group; Sop: scopolamine model group; Riv: rivastigmine positive control group; VD: vitamin D group—mice in this group were fed a vitamin D-enriched diet; VD + VA: vitamin D + vitamin A group—mice in this group were fed a vitamin D + vitamin A-enriched diet; P: sunflower lecithin group—mice in this group were fed a sunflower lecithin-enriched diet; P + VA: sunflower lecithin + vitamin A group—mice in this group were fed a sunflower lecithin + vitamin A-enriched diet. The specific amounts of the functional factors mentioned above are shown in Table 1.

**Table 4:** The numbers of differentially expressed genes in the normal group and intervention group were significantly different from that in the model group.

Group	Upregulated Gene Number	Downregulated Gene Number
Con	326	640
VD	172	144
VD + VA	319	228
P	555	363
P + VA	485	216

## Data Availability

All the data are available from the first author upon reasonable request.

## Supplementary Materials

The following supporting information can be downloaded at: https://www.mdpi.com/article/10.3390/nu17030553/s1, Figure S1: Chemical structures of Vitamin D3 and Vitamin A, Acetate; Table S1: The composition ratio of sunflower lecithin components.
