# Supplementary material for: Sunflower Oil Fortified with Vitamins D and A and Sunflower Lecithin Ameliorated Scopolamine-Induced Cognitive Dysfunction in Mice and Exploration of the Underlying Protective Pathways
Source: Nutrients. 2025 Jan 31;17(3):553. doi: 10.3390/nu17030553 (PMC11819866; doi:10.3390/nu17030553)
Supplement: Supplementary file 1 [file nutrients-17-00553-s001.zip › nutrients-3439188-supplementary.pdf]

Supplementary materials

Table S1. The composition ratio of sunflower lecithin components.

	sunflower lecithin
Phosphatidylcholine (PC)	42%
Phosphatidylinositol (PI)	36%
Phosphatidylethanolamine (PE)	15%

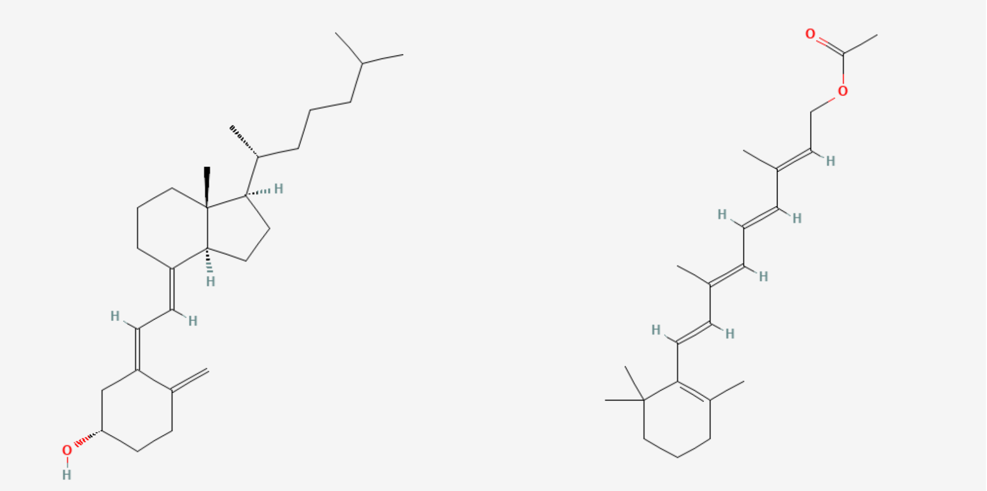

Vitamin D3 Vitamin A, Acetate

Figure S1. Chemical structures of Vitamin D3 and Vitamin A, Acetate.
